# Bidirectional relationship between child happiness and sleep quality and the predictive role of prenatal psychosocial support: results from U.S. and China cohort studies

**DOI:** 10.1007/s12519-025-00903-9

**Published:** 2025-05-30

**Authors:** Ying Dai, Naixue Cui, Xiaopeng Ji, Adrian Raine, Therese S. Richmond, Jianghong Liu

**Affiliations:** 1 School of Nursing and Medicine, University of Pennsylvania, 418 Curie Blvd., Room 426, Claire M. Fagin Hall, Philadelphia, PA 19104-6096 USA; 2 School of Nursing and Rehabilitation, Shandong University, Jinan, China; 3 School of Nursing, College of Health Sciences, University of Delaware, Newark, USA; 4 Department of Criminology, Psychiatry, and Psychology, University of Pennsylvania, Philadelphia, PA USA

**Keywords:** Child, Happiness, Maternal, Psychosocial support, Sleep quality

## Abstract

**Background:**

The relationship between daily affective states and subjective sleep measures is known to be reciprocal in both adults and children. However, its consistency across infancy to early adolescence and varying sociocultural contexts remain unclear. We investigated the bidirectional relationship between happiness and sleep quality in children from the U.S. and China and examined the predictive role of maternal prenatal psychosocial support.

**Methods:**

A total of 1300 children aged 11–12 years in the U.S. Healthy Brain and Behavior Study (HBBS) and China Jintan Child Cohort (CJCC) studies were included in the analyses. Happiness and sleep quality from infancy to early adolescence were retrospectively reported by mothers. Prenatal psychosocial factors were recalled by mothers through a structured interview. Random-intercept cross-lagged panel modeling was conducted to analyze the data.

**Results:**

Happiness and sleep quality trajectories differed significantly between U.S. adolescents in the HBBS and Chinese adolescents in the CJCC (*P* < 0.001). In both cohorts, cross-lagged effects showed consistent associations between happiness and subsequent sleep quality across the five time points (coefficients: 0.086–0.154, *P* < 0.01). The relationship between sleep quality and subsequent happiness was significant from 1–3 to 11 years. The bidirectional relationship was stronger in the Chinese cohort. Maternal prenatal psychosocial support positively predicted children’s happiness (*β* = 0.072, *P* < 0.001) and sleep quality (*β* = 0.056, *P* < 0.001) trajectories.

**Conclusions:**

A bidirectional relationship exists between children’s happiness and sleep quality, with potential cultural variations. Maternal prenatal psychosocial support plays a key role in fostering children’s long-term emotional well-being and sleep quality.

**Graphical abstract:**

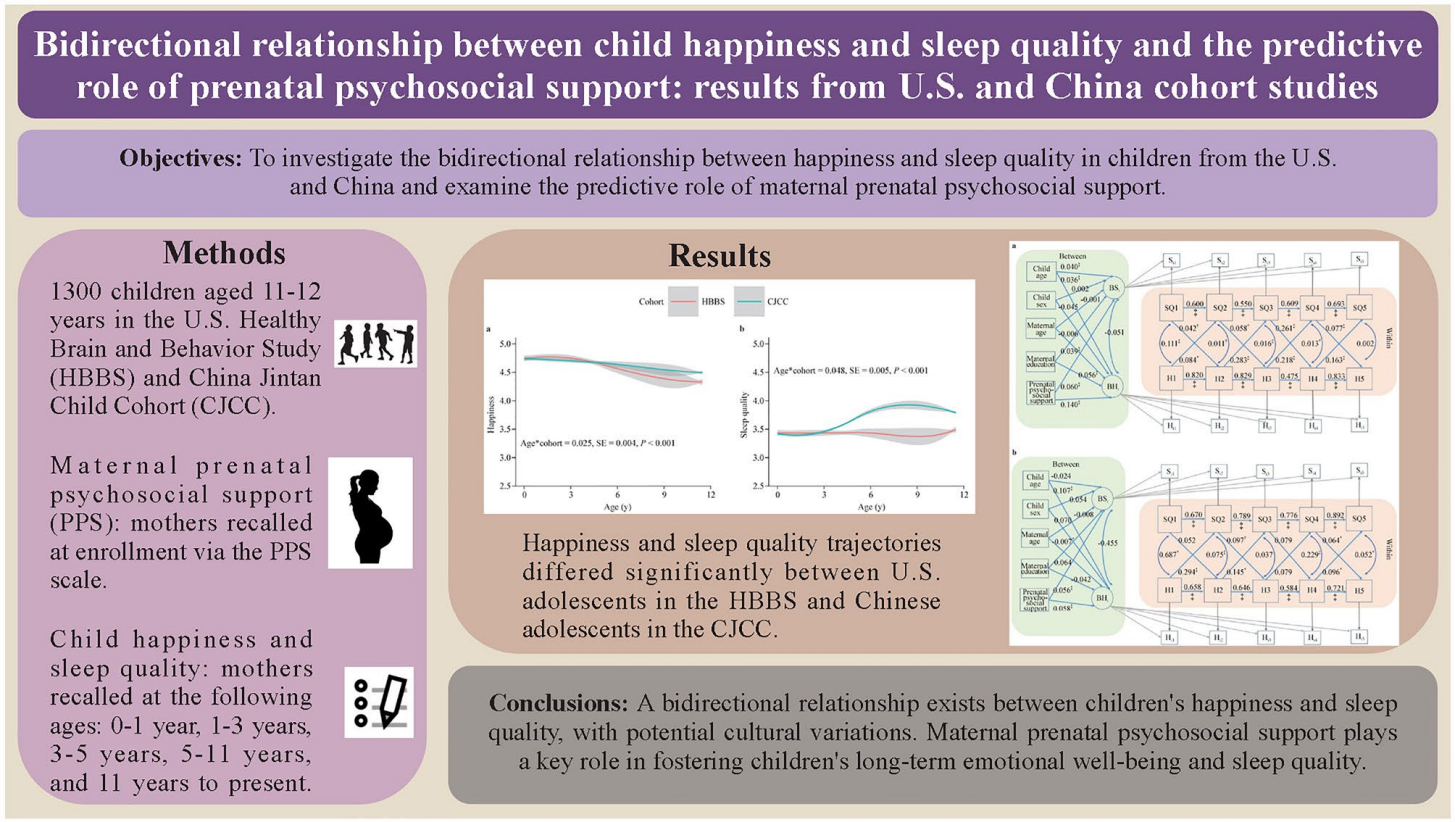

**Supplementary Information:**

The online version contains supplementary material available at 10.1007/s12519-025-00903-9.

## Introduction

Sleep health is a vital component of human physical health and mental wellbeing [1]. Poor sleep quality is associated with poor school performance in children and adolescents [2], behavioral problems [3], emotional overeating and greater food responsiveness, which can contribute to obesity [4], and subjective wellbeing [5]. Likewise, positive affect, such as happiness, is essential for health and well-being. Happiness refers to the subjective feeling of well-being, consisting of one’s life satisfaction, positive emotions such as feeling happy and low levels of negative affect such as sadness [6]. For adults, increased happiness is associated with decreased risks of all-cause mortality and various chronic illnesses [7] and is a protective factor for sleep, such as adequate sleep duration [8]. In adolescents, greater happiness is associated with healthy lifestyles, such as healthy eating habits and daily physical activity, and fewer risk behaviors, such as smoking or drinking [9, 10]. Childhood happiness may have a long-term effect on children’s cognitive development and education attainment [11] and workplace success in adulthood [12]. However, the understanding of how sleep quality and happiness change across child and early adolescence developmental stages is limited [13].

Affect is closely correlated with sleep, and this relationship is potentially bidirectional [14]. A systematic review revealed an overall reciprocal relationship between daily affective states and subjective sleep measures in both adults and children [15]. Specifically, daytime positive affect (e.g., happiness and joy) predicts the next day’s better nighttime sleep; conversely, self-reported sleep quality is positively associated with the next day’s positive affect and inversely correlated with the next day’s negative affect (e.g., worry, sadness and anger) in both children and adolescents [15]. It remains unclear whether this relationship holds true across children’s long-term development course. Furthermore, while two systematic reviews have documented a reciprocal relationship between sleep and negative emotions and externalizing behaviors [16, 17], few studies have investigated the relationship between positive emotions and sleep. Recent studies revealed that greater self-reported happiness in adolescents was associated with fewer sleep problems and better sleep quality [18, 19]. However, these studies employed a cross-sectional design and included only one child’s developmental age, leaving the directionality of the relationship between happiness and sleep quality across early childhood to adolescence largely unexplored.

Happiness and sleep quality, both shaped by subjective perceptions, may differ significantly across cultures [20, 21]. For example, Americans generally take an “incremental” perspective toward happiness where they perceive that positive emotions will induce more positive outcomes, whereas Chinese culture takes a “dialectical” lens to view happiness and believes that good things follow from bad things and that misfortune can come from happiness [22]. Similarly, people’s sleep practices and attitudes toward sleep vary across different cultures. A study revealed that, compared with European Canadians, Japanese university students reported significantly less sleep duration and sleep efficiency, yet they reported less tiredness [23]. With respect to children, parental attitudes toward their children’s sleep, sleep arrangements, and perceptions of optimal sleep vary across different cultures [24]. Given the culturally specific nature of happiness and sleep, studies comparing the relationship between happiness and sleep in children across different cultures are warranted.

Among the wide range of potential factors that influence happiness and sleep health, social support, especially maternal perceived psychosocial support during pregnancy, stands out as a promising protective factor. Maternal stress and inadequate psychosocial support during pregnancy have been associated with alterations in fetal brain development, potentially leading to sleep disturbances and emotional dysregulation in children [25]. Research indicates that children whose mothers receive strong psychosocial support during pregnancy tend to exhibit higher levels of happiness and better sleep patterns as they grow [26]. These findings suggest that maternal support during pregnancy not only buffers against prenatal stress but also lays the foundation for healthier emotional and behavioral outcomes in children. Despite these findings, there is still a limited understanding of how maternal psychosocial support during pregnancy continues to affect a child’s sleep and happiness trajectories across different developmental stages. Investigating this relationship is vital for developing targeted interventions that promote maternal well-being and, by extension, foster healthier outcomes for children.

This study explores the bidirectional relationship between sleep and happiness across different stages of child development and different cultural contexts while examining the predictive role of maternal perceived psychosocial support during pregnancy in shaping these developmental changes over time.

The bidirectional relationship between sleep and happiness is a key focus, with the hypothesis that these two factors influence each other throughout childhood and adolescence. The study also considers cultural differences, expecting the relationship between sleep and happiness to vary between Chinese and U.S. contexts, reflecting diverse cultural attitudes and practices. Additionally, maternal psychosocial support during pregnancy is proposed as a predictor of both sleep quality and happiness in children, potentially shaping their developmental trajectories over time.

Specifically, this study aimed to examine whether a bidirectional relationship exists between happiness and sleep quality across children’s different developmental stages, whether this relationship differs across the Chinese and U.S. cultural contexts, and whether maternal psychosocial support during pregnancy predicts this bidirectional relationship.

## Methods

### Participants

Participants for this study were drawn from two cohorts: the China Jintan Child Cohort (CJCC) study [27, 28] and the U.S. Healthy Brain and Behavior Study (HBBS) [29]. Detailed descriptions of these two cohorts have been described elsewhere [28, 29]. Briefly, the CJCC study employed a stratified random sampling of four preschools in Jintan city to represent varying socioeconomic statuses. A total of 1656 children aged 3–5 years were recruited to explore a wide range of environmental factors influencing children’s health and behavior development [27]. These children are followed through adolescence.

The U.S. HBBS consists of 454 adolescents aged 11–12 years (mean age = 11.92, SD = 0.60) who reside in urban or suburban communities in Philadelphia [29]. The HBBS aims to first investigate the environmental, biological, and psychosocial risk factors for early adolescent behavior problems; second, conduct a clinical trial to test the effectiveness of nutritional and cognitive-behavioral treatment on a subsample of children with significant aggressive behavior; and third, conduct a long-term follow-up on young adulthood behavior and health outcomes. The HBBS recruitment focused on geographic areas within Philadelphia County and nearby suburbs with substantial populations of 11–12-year-olds (≥ 400 children per zip code), as identified in the 2000 Census. Participants were recruited through targeted mailings to parents via lists from a local children’s hospital primary care network, community outreach, and participant referrals. The resulting sample (80.3% African American) reflects the demographic composition of the urban and inner-suburban Philadelphia communities from which participants were recruited rather than the broader U.S. population. Institutional Review Board (IRB) approval was obtained from the University of Pennsylvania, the Ethical Committee for Research at Jintan Hospital in China, and the IRB of the city of Philadelphia. All parents signed informed consent, and children (who were of school age) gave verbal assent to participate in the study.

### Measures

For both cohorts, mothers provided retrospective reports on their children’s happiness and sleep quality at ages 10–12 years. For the CJCC cohort, data were collected from 2010–2011. Among the 956 mothers contacted, 846 completed and returned a structured questionnaire about their pregnancy history, life events, psychosocial support, and child’s sleep and happiness. For the HBBS cohort, data were collected from 2009–2012. All 454 mothers participated, providing similar information through structured interviews during the baseline risk assessment for behavior problems.

#### Children’s happiness

During the second wave of CJCC data collection and the baseline risk assessment stage of the HBBS (when children were 10–12 years old), mothers provided retrospective assessments of their children’s happiness across different age periods. They respond to a single question: “in general, how happy do you think your child was during the following years: 0–1 year, 1–3 years, 3–5 years, 5–11 years, and 11 years to present?” with the following answers: 1 = “very unhappy”, 2 = “unhappy”, 3 = “neither happy or unhappy”, 4 = “fairly happy”, and 5 = “very happy”.

#### Children’s sleep quality

Similarly, at the same assessment point, mothers provided retrospective ratings of their children’s sleep quality across the same age periods. They responded to the following question: “in general, how was your child’s sleep quality during the following ages: 0–1 year, 1–3 years, 3–5 years, 5–11 years, and 11 years to present?”, with 1 = “poor”, 2 = “fair”, 3 = “good”, and 4 = “very good”.

#### Maternal psychosocial support during pregnancy

Maternal psychosocial support during pregnancy in both cohorts was measured via the prenatal psychosocial scale (PPS), which was developed simultaneously in English and Chinese by the principal investigator for the CJCC, who also served as the coinvestigator for the HBBS, to enable cross-cultural assessment. The PPS includes ten items assessing various dimensions of the prenatal experience, such as pregnancy planning (e.g., "was this pregnancy planned?"), emotional responses to pregnancy (e.g., "how happy did the pregnancy make you?" and "how happy was your partner about your pregnancy after he found out?"), and social support (e.g., "how supportive were your family and friends during your pregnancy?"). Other items capture potentially adverse experiences, such as thoughts or actions related to pregnancy termination and postpartum depression. Responses are primarily scored on dichotomous (yes/no) or ordinal scales (e.g., "not at all", "somewhat", and "very"). The sum of the ten items yields a total PPS score, with higher scores indicating lower maternal stress and better perceived support. The scale is treated as a continuous measure without predetermined cutoff points. The PPS demonstrated good internal consistency, with a Cronbach’s *α* of 0.750 in the CJCC and 0.708 in the HBBS.

### Covariates

Children’s sex, race (only collected from the HBBS), maternal age when the child was born, and maternal education level (in years) were considered potential covariates on the basis of the previous literature [30, 31] and were included in the data analysis. All covariates were collected at the recruitment phase of the two studies.

### Data analysis

Descriptive analysis was used to assess the characteristics of the study sample. Spearman correlations were used to examine the relationships between child happiness and sleep quality over time. The binary variable, child sex, was coded numerically (e.g., 1 = male, 0 = female) for the purpose of conducting Spearman correlation analyses.

Two-level mixed-effects linear modeling was used to examine the trajectories of child sleep quality and happiness across age periods and test whether these trajectories differed between the CJCC and HBBS cohorts. Child age was entered as the level 1 variable, whereas cohort membership and other covariates were included as level 2 variables. The interaction term “age*cohort” was used to test whether sleep quality and happiness trajectories were significantly different between cohorts. The model can be described as follows:

$${\text{Level }}1{:}\;Y_{ij} = \beta_{0i} + \beta_{1i} (age_{ij} ) + \beta_{2i} \left( {U_{ij} } \right) + \beta_{3i} (age_{ij} * U_{ij} ) + \varepsilon_{ij}$$

$$\begin{aligned} {\text{Level }}2:\; & \beta_{0i} = \beta_{0} + \alpha_{i} \\ & \beta_{1i} = \beta_{1} + \gamma_{i} \\ & \beta_{2i} = \beta_{2} \\ & \beta_{3i} = \beta_{3} \\ \end{aligned}$$

The composite model can be written as:

$$Y_{ij} = \beta_{0} + \beta_{1} (age_{ij} ) + \beta_{2} (U_{ij} ) \, + \beta_{3} (age_{ij} *U_{ij} ) + \alpha_{i} + \gamma_{i} (age_{ij} ) + \varepsilon_{ij}$$

where *Y*_*ij*_ refers to sleep quality or happiness for child* i* at time *j*. Age refers to the child age period, U refers to cohort membership (CJCC vs. HBBS), and “*age* * *U*” refers to the interaction between child age and cohort. *β*_0_ is the global intercept, and *β*_1_ and *β*_2_ are the fixed effects for child age and the explanatory variable/covariate, respectively. *α*_*i*_, *γ*_*i*_*,* and *ε*_*ij*_ are the random effects of the composite model. *α*_*i*_ and *γ*_*i*_ follow the multivariate normal distribution. The interaction term (*β*_3_) was used to test whether sleep quality and happiness trajectories were significantly different between the CJCC and the HBBS. Mixed-effects linear modeling with maximum likelihood estimation was conducted with the “lmerTest” package [32].

Random-intercepts cross-lagged panel modeling (RI-CLPM) was used to estimate the reciprocal relationship between happiness and sleep quality across time and further explore whether observed differences in children’s happiness and sleep quality across time were predicted by prenatal maternal psychosocial support [33]. The advantage of the RI-CLPM is that the RI-CLPM does not make the “strong” assumption that all individuals’ observed measures vary around a common group mean over time [33]. Instead, the RI-CLPM assumes that each individual has his/her own trait-like characteristics and that the observed measures fluctuate around these unique trait-like means [34]. The equations of the RI-CLPM can be written as:

$${x}_{it} = {\mu }_{t} + {\kappa }_{i} + {p}_{it}^{*} ,$$

$${y}_{it} = {\pi }_{t} + {\omega }_{i} + {q}_{it}^{*} ,$$

where ${x}_{it}$ refers to the observed value of happiness for child *i* at time *t*, ${y}_{it}$ refers to the observed value of sleep quality for child *i* at time *t*, ${\mu }_{t}$ and ${\pi }_{t}$ are the group means of happiness and sleep quality, respectively, and ${\kappa }_{i}$ and ${\omega }_{i}$ are the individual’s trait-like fluctuations from the group means of happiness and sleep quality, respectively. ${p}_{it}^{*}$ and ${q}_{it}^{*}$ refer to individual *I*’s temporal deviations from his/her expected scores in terms of happiness and sleep quality at time *t*, respectively, and can be further expressed as:

$${p}_{it}^{*} = {\alpha }_{t}^{*}{p}_{i,t-1}^{*} + {\beta }_{t}^{*}{q}_{i,t-1}^{*} + {\vartheta }_{it}^{*} ,$$

$${q}_{it}^{*} = {\delta }_{t}^{*}{q}_{i,t-1}^{*} + {\gamma }_{t}^{*}{p}_{i,t-1}^{*} + {\upsilon }_{it}^{*} ,$$

where ${\alpha }_{t}^{*}$ and ${\delta }_{t}^{*}$ represent the within-person autocorrelation effects of happiness and sleep quality, respectively. A positive effect in ${\alpha }_{t}^{*}$ indicates that an increase in one’s observed score in his/her expected happiness score is associated with a further increase in his/her observed happiness score (or sleep quality score) in the next occasion. Similarly, a positive ${\delta }_{t}^{*}$ coefficient indicates that an increase in one’s observed sleep quality score is associated with a further increase in his/her observed sleep quality score in the next occasion. ${\beta }_{t}^{*}$ and ${\gamma }_{t}^{*}$ represent the cross-lagged effects of happiness and sleep quality, respectively, and are the main focus of the RI-CLPM. Specifically, ${\beta }_{t}^{*}$ indicates the extent to which an individual’s change in happiness at time t can be predicted from the individual’s prior fluctuation in his/her expected score in sleep quality at time *t* − 1, adjusting for the individual’s happiness carry-over effects and the concurrent correlation between happiness and sleep. Similarly, ${\gamma }_{t}^{*}$ represents the extent to which one’s sleep quality at time *t* can be predicted from his/her happiness at time *t* − 1, controlling for his/her sleep quality autocorrelation effects and the concurrent correlation between sleep quality and happiness. ${\vartheta }_{it}^{*}$ and ${\upsilon }_{it}^{*}$ represent the time-specific residual components for the within-person deviations in happiness (${p}_{it}^{*}$) and sleep quality (${q}_{it}^{*}$), respectively. They capture the unexplained variance in these deviations after accounting for carry-over (${\alpha }_{t}^{*}$, ${\delta }_{t}^{*}$) and spill-over effects (${\beta }_{t}^{*}$, ${\gamma }_{t}^{*}$). These residual terms are assumed to have a mean of zero and are uncorrelated with the predictors (${p}_{i,t-1}^{*}$ and ${q}_{i,t-1}^{*}$), ensuring unbiased parameter estimation.

The concurrent correlation between happiness and sleep quality is adjusted in the RI-CLPM by explicitly modeling the covariance between the residual terms ${\vartheta }_{it}^{*}$ and ${\upsilon }_{it}^{*}$. This adjustment accounts for any shared variance at a given time point that is not explained by the specified carry-over and spill-over effects, thereby improving the precision of the estimated relationships. Furthermore, maternal psychosocial support and other covariates were included as time-invariant predictors of the between-level variations in happiness and sleep quality. To measure nuanced changes in happiness and sleep quality across different developmental ages, we did not impose equal constraints on the spillover effects, carry-over effects, or concurrent correlations.

Multi-group analysis was further conducted to test whether the relationship between happiness and sleep quality has different patterns between the CJCC and the HBBS. Following Mund and Hamaker’s suggestions [35], we first developed a basic multi-group model without constraining the autoregressive or cross-lagged parameters. Next, we constrained the regression coefficients to be identical between the two cohorts. The Chi-square difference test (Δχ^2^) was used to assess whether the baseline multi-group model and the constrained model were significantly different.

To explore whether the model fits the data well, the following model fit indices and cutoff criteria were used: comparative fit index > 0.95; Tucker–Lewis index > 0.95; root-mean-square error of approximation < 0.06; and standardized root-mean-square residual < 0.08 [36]. With respect to missing values, 2.0%–6.0% of the children in the CJCC study had missing values for their demographic information, and 46.6% of the children had missing values for their sleep quality or happiness across childhood. In the HBBS, 2.9%–9.3% of the children had missing values for their demographic information or prenatal maternal psychosocial support score, and less than 2.5% of the children had missing values for their happiness or sleep quality score. Our preliminary analysis revealed that the missing data in both cohorts were random (Supplementary Figs. 1 and 2). Missing values in both cohorts were imputed with the missForest package [37]. This package imputes missing values via the random forest algorithm with no assumption of a certain distribution of variables [37]. To enhance the robustness of the statistical analysis, the RI-CLPM was conducted with both imputed and non-imputed data, and the results were compared. All tests were two-tailed, and *P* < 0.05 was considered statistically significant. All the statistical analyses were conducted with R (version 4.4.0). The RI-CLPM was conducted with the Lavaan package [38].

## Results

### Sample characteristics

Table 1 shows the participants’ demographic information for both cohorts. Compared with American children in the HBBS, Chinese children in the CJCC were younger when they were surveyed, and their male proportion was slightly greater. Mothers of Chinese children in the CJCC received less education and reported receiving less psychosocial support during their pregnancy. Approximately 80% of American children in the HBBS were African American, with 13% being Caucasian.

**Table 1:** Participants’ demographic characteristics

Variables	CJCC (*n* = 846)	HBBS (*n* = 454)	T/*χ*^2^ test	*P*
Child information				
Child age at survey (y), mean (SD)	11.36 (0.79)	11.92 (0.60)	− 14.5	< 0.001
Sex, *n* (%)			0.2	0.7
Male	444 (52.5)	230 (51.0)		
Female	402 (47.5)	221 (49.0)		
Ethnicity, *n* (%)				
Caucasian/White		54 (12.0)		
African American		362 (80.3)		
Hispanic/Latino		4 (0.9)		
Multiracial		22 (4.9)		
Other		9 (2.0)		
Parental information, mean (SD)				
Maternal age when child was born	25.89 (2.87)	26.40 (7.07)	− 3.52	< 0.001
Maternal education in years	10.74 (2.97)	13.40 (2.25)	− 39.6	< 0.001
Maternal psychosocial stress score during pregnancy	12.65 (1.08)	13.44 (2.84)	− 5.69	< 0.001
Prenatal life events, *n* (%)				
Divorce/separation	3 (0.4)	60 (13.2)	104	< 0.001
Fired from job	17 (2.0)	94 (20.7)	131	< 0.001
Moving	33 (3.9)	137 (30.2)	179	< 0.001
Financial problems	38 (4.5)	148 (32.6)	190	< 0.001
Accident	5 (0.6)	21 (4.6)	23	< 0.001
Conflict with significant other	13 (1.5)	164 (36.1)	300	< 0.001
Illness or death of relatives/friends/significant other	26 (3.0)	73 (16.1)	70	< 0.001

*CJCC* China Jintan Child Cohort study, *HBBS* Healthy Brain and Behavior Study, *SD* standard deviation

**Fig. 1:**
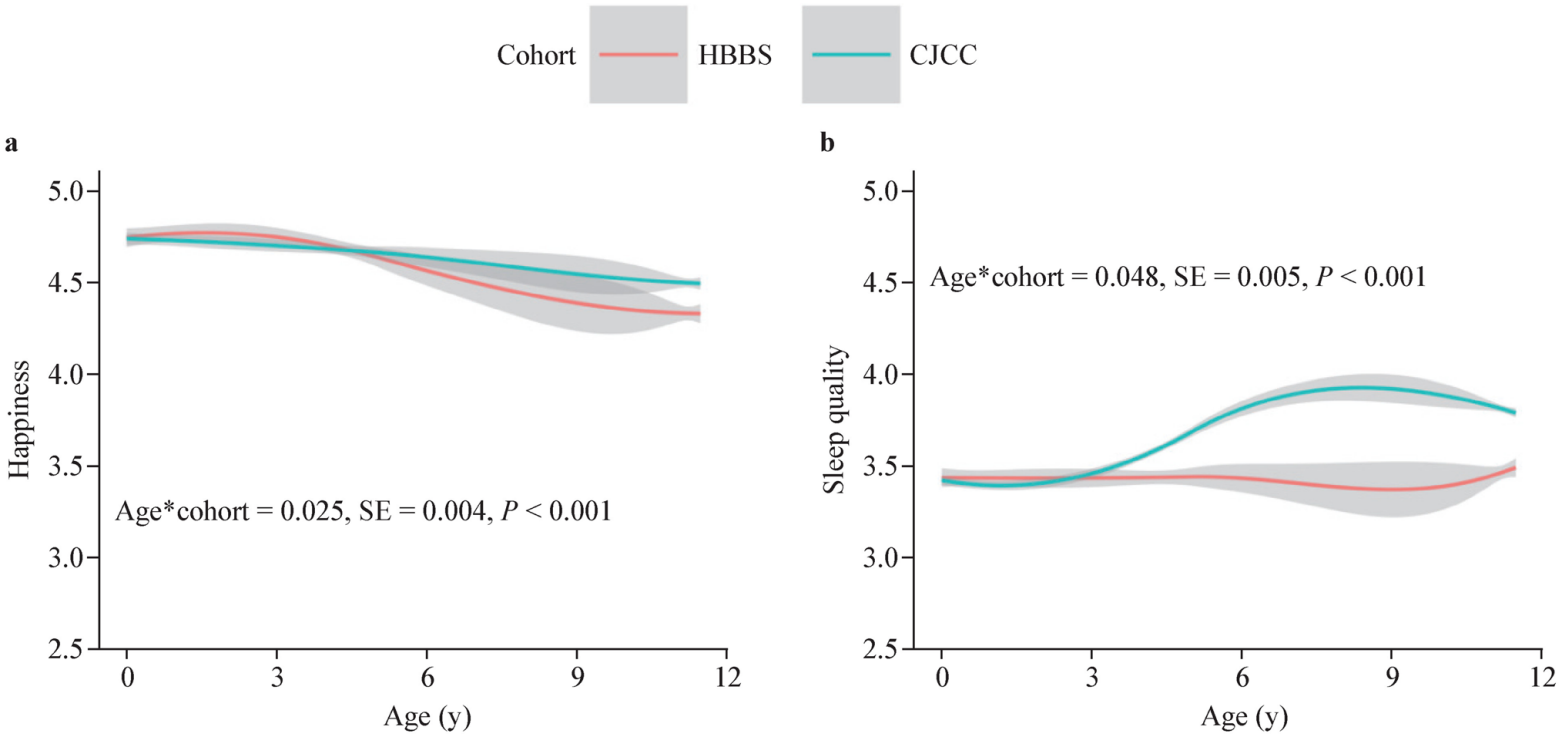
Children’s average happiness trajectories (**a**) and average sleep quality trajectories (**b**) over time. *HBBS* Healthy Brain and Behavior Study, *CJCC* China Jintan Child Cohort, *SE* standard error

Regarding the happiness trajectory, the happiness trajectory of the CJCC children followed an overall stable path, with a minor decreasing trend over time, whereas the happiness score of the children in the HBBS increased from 0–3 years, followed by a decrease from 3–11.5 years of age (Fig. 1a). The happiness scores of both cohorts of children were comparable when they were 0–3 years of age. After three years of age, children in the CJCC had higher happiness scores than did children in the HBBS. Mixed-effects modeling revealed that the interaction term “age*cohort” for the happiness trajectory was statistically significant [*β* = 0.025, standard error (SE) = 0.004, *P* < 0.001; Supplementary Table 1, model 2], indicating significant differences in happiness trajectories between CJCC and HBBS children.

Compared with children in the HBBS, children in the CJCC had significantly higher sleep quality scores from infancy to preadolescence. Furthermore, children from both cohorts had increased sleep quality trajectories from 0–5 years. After five years of age, the sleep quality trajectory of CJCC children improved from 5–11 years, followed by a minor decrease after 11 years of age. However, the sleep quality trajectory of children in the HBBS decreased after five years of age (Fig. 1b). Mixed-effects modeling indicated that the interaction term “age*cohort” for the sleep quality trajectory was statistically significant (*β* = 0.048, SE = 0.005, *P* < 0.001; Supplementary Table 1, model 4), indicating notable differences in sleep quality trajectories between children in the CJCC and HBBS cohorts.

### Correlations between child happiness and sleep quality

Child happiness and child sleep quality were highly correlated across time points (Table 2). The autocorrelations of happiness (i.e., the correlation of happiness between two successive time points) ranged between 0.582 and 0.818, which were greater than their concurrent correlations with sleep quality (ranging between 0.263 and 0.372). Similarly, the autocorrelations of sleep quality (ranging between 0.339 and 0.855) were greater than their concurrent correlations with happiness. Furthermore, children’s age, sex, cohort, maternal age, maternal education, and maternal prenatal psychosocial support were all significantly correlated with children’s happiness and sleep quality and thus were entered as between-person predictors in later RI-CLPMs.

**Table 2:** Correlation between sex, cohort, maternal psychosocial support, child sleep quality and child happiness across time

No.	Variables	1	2	3	4	5	6	7	8	9	10	11	12	13	14	15
1	Cohort															
2	Child age	− 0.369^‡^														
3	Child sex	− 0.669^‡^	0.291^‡^													
4	Maternal education	− 0.400^‡^	0.116^‡^	0.294^‡^												
5	Maternal age	0.011	0.008	− 0.017	0.116^‡^											
6	Maternal PPS	− 0.278^‡^	0.043	0.159^‡^	0.180^‡^	0.228^‡^										
7	Happiness at t1	− 0.267^‡^	0.031	0.173^‡^	0.177^‡^	− 0.039	0.322^‡^									
8	Happiness at t2	− 0.291^‡^	0.053	0.210^‡^	0.152^‡^	− 0.022	0.309^‡^	0.854^‡^								
9	Happiness at t3	− 0.016^†^	0.013	0.089^†^	0.078^†^	0.051	0.283^‡^	0.694^‡^	0.776^‡^							
10	Happiness at t4	0.150^‡^	− 0.082^†^	− 0.100^‡^	− 0.050	0.013	0.189^‡^	0.391^‡^	0.412^‡^	0.580^‡^						
11	Happiness at t5	0.137^‡^	− 0.037	− 0.137^‡^	− 0.085^†^	0.010	0.175^‡^	0.365^‡^	0.363^‡^	0.490^‡^	0.821^‡^					
12	Sleep quality at t1	0.187^‡^	− 0.112^‡^	− 0.080^‡^	0.014	0.011	0.032	0.262^‡^	0.210^‡^	0.218^‡^	0.180^‡^	0.144^‡^				
13	Sleep quality at t2	0.147^‡^	− 0.144^‡^	− 0.117^‡^	0.063*	0.066*	0.112^‡^	0.283^‡^	0.256^‡^	0.248^‡^	0.200^‡^	0.182^‡^	0.726^‡^			
14	Sleep quality at t3	0.221^‡^	− 0.165^‡^	− 0.168^‡^	− 0.006	0.035	0.084^†^	0.249^‡^	0.237^‡^	0.236^‡^	0.239^‡^	0.213^‡^	0.624^‡^	0.772^‡^		
15	Sleep quality at t4	0.283^‡^	− 0.189^‡^	− 0.228^‡^	− 0.077^‡^	0.048	0.087^†^	0.172^‡^	0.159^‡^	0.205^‡^	0.304^‡^	0.292^‡^	0.436^‡^	0.576^‡^	0.752^‡^	
16	Sleep quality at t5	0.236^‡^	− 0.074^†^	− 0.172^‡^	− 0.062^‡^	− 0.002	0.091^†^	0.134^‡^	0.139^‡^	0.193^‡^	0.331^‡^	0.319^‡^	0.367^‡^	0.492^‡^	0.634^‡^	0.816^‡^

Time points: t1: 0–1 year, t2: 1–3 years, t3: 3–5 years, t4: 5–11 years, t5: 11 years–present. *PPS* prenatal psychosocial support. Spearman correlation was conducted: ^*^P < 0.05, ^†^*P* < 0.01, ^‡^*P* < 0.001

### Bidirectional relationship between child happiness and sleep quality

A total of six RI-CLPM models were built to explore the relationship between child happiness and sleep quality over time. We first combined the two cohorts (providing a larger sample size with greater power) to explore the bidirectional relationship. Next, we separately analyzed the CJCC and the HBBS to evaluate whether the bidirectional relationship holds in the two cohorts. All the models generally had good model fit indices (Table 3).

**Table 3:** Model fit indices of random-intercepts cross-lagged modeling

Variables	*χ*^2^/*df*	CFI	TLI	RMSEA (90% CI)	SRMR	Δ*χ*^2^
No between-person covariates						
CJCC + HBBS	92.389/22 = 4.20	0.991	0.981	0.048 (0.021, 0.073)	0.023	
CJCC	93.622/21 = 4.46	0.991	0.981	0.064 (0.051, 0.077)	0.023	
HBBS	50.107/22 = 2.28	0.987	0.974	0.053 (0.034, 0.073)	0.026	
Multi-group analysis						
Unconstrained model	386.263/52 = 7.43	0.967	0.942	0.069 (0.062, 0.076)	0.109	
Regression weights constrained model	485.486/68 = 7.14	0.945	0.927	0.063 (0.057, 0.069)	0.106	99.2 (16)^‡^
Add between-person covariates						
CJCC + HBBS	371.509/70 = 5.31	0.967	0.951	0.058 (0.052, 0.063)	0.052	
CJCC	337.025/62 = 5.44	0.968	0.951	0.072 (0.065, 0.080)	0.050	
HBBS	167.859/85 = 1.97	0.964	0.056	0.046 (0.036, 0.057)	0.055	
Multi-group analysis						
Unconstrained model	521.673/132 = 3.95	0.963	0.946	0.067 (0.061, 0.074)	0.053	
Regression weights constrained model	609.488/148 = 4.12	0.956	0.943	0.069 (0.064, 0.075)	0.058	87.8 (16)^‡^

Between-person covariates: child sex, child age at survey, maternal education level, maternal age when child was born, and prenatal psychosocial support. Regression weights constrained model: both the autoregressive regression coefficients and the cross-lagged coefficients were constrained to be equal between CJCC and HBBS. *CFI* comparative fit index, *TLI* Tucker–Lewis index, *RMSEA* root-mean-square error of approximation, *SRMR* standardized root-mean-square residual. ^‡^*P* < 0.001

Using the combined two cohorts, we examined the extent to which the variance in happiness and sleep quality was attributed to between-person and within-person components. The between-person variances of the happiness and sleep quality intercepts were 0.016 (SE = 0.029, *P* = 0.591) and 0 (SE = 0.103, *P* = 0.272), respectively. The within-person variance of the latent residuals ranged between 0.111 (SE = 0.005, *P* < 0.001, 1–3 years) and 0.345 (SE = 0.032, *P* < 0.001, 0–1 year) for happiness and between 0.227 (SE = 0.007, *P* < 0.001, 10–11.5 years) and 0.892 (SE = 0.109, 0–1 year) for sleep quality. Both the variances in happiness and sleep quality across time were attributable mostly to within-person variation; thus, traditional cross-lagged modeling may be sufficient. However, the RI-CLPM was maintained since the traditional cross-lagged model is nested in the RI-CLPM, which further allows each child’s happiness and sleep quality to fluctuate around his or her own trait-like happiness and sleep quality scores.

**Fig. 2:**
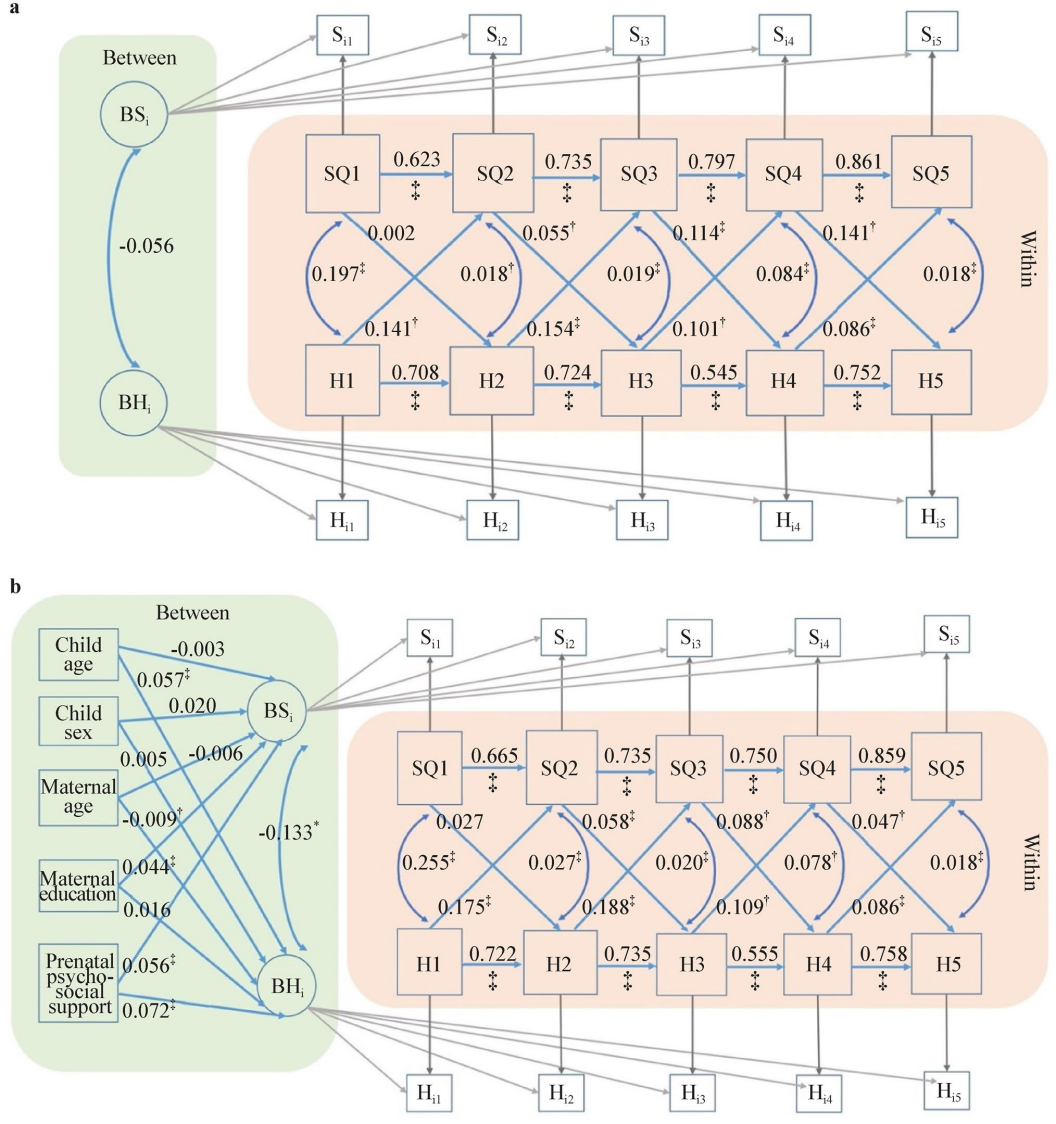
Random intercepts cross-lagged panel modeling of the combined CJCC and HBBS cohorts. **a** Without between-person predictors; **b** with between-person predictors. *HBBS* Healthy Brain and Behavior Study, *CJCC* China Jintan Child Cohort, *SQ* sleep quality, *H* happiness, *BSi* between-person random intercepts for sleep quality across five occasions, *BHi* between-person random intercepts for happiness across five occasions. ^*^P < 0.05, ^†^*P* < 0.01, ^‡^*P* < 0.001

The results of the RI-CLPM with and without time-invariant predictors are shown in Fig. 2. Figure 2a shows that the cross-lagged effects of happiness at time *t* on sleep quality at time *t* + 1 remain statistically significant across all five time occasions, with the standard regression coefficients $\beta$ ranging between 0.086 and 0.154, all *P* < 0.01, suggesting that a possible deviation above one’s person-specific mean in happiness at an earlier time is associated with a subsequent elevation in the person’s specific mean in sleep quality, controlling for previous deviations from the person-specific mean in sleep quality and the concurrent within-time correlation between sleep quality and happiness. On the other hand, the effects of sleep quality at time *t* on happiness at time *t* + 1 remain statistically significant only from time 2 (1–3 years) to time 5 (11 years to present), indicating that the cross-lagged effects of sleep quality on happiness were significant only from toddlerhood to early adolescence.

Figure 2b shows that the cross-lagged effects of happiness on sleep quality and vice versa still remain statistically significant after introducing between-person time-invariant predictors, including cohort, child age, child sex, maternal age when the child was born, maternal education, and maternal psychosocial support during pregnancy. Among these time-invariant predictors, maternal psychosocial support during pregnancy was positively associated with the random intercepts for children’s happiness trajectory ($\beta$ = 0.072, SE = 0.007, *P* < 0.001) and sleep quality trajectory ($\beta$ = 0.056, SE = 0.009, *P* < 0.001), suggesting that greater psychosocial support during pregnancy predicts children’s average level of happiness and better sleep quality from infancy to early preadolescence.

**Fig. 3:**
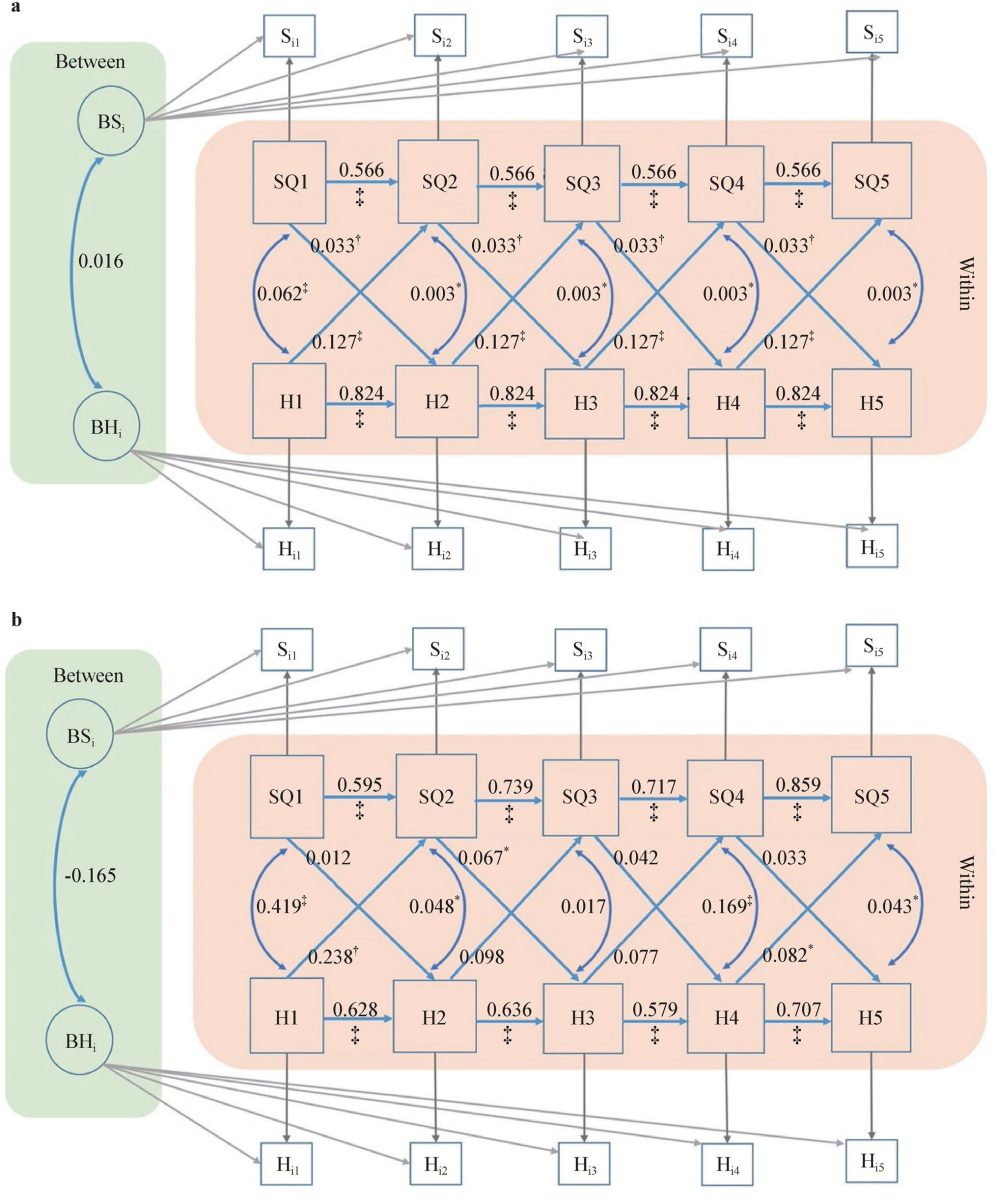
Random intercepts cross-lagged panel modeling without between-person covariates for CJCC (**a**) and HBBS (**b**). *HBBS* Healthy Brain and Behavior Study, *CJCC* China Jintan Child Cohort, *SQ* sleep quality, *H* happiness, *BSi* between-person random intercepts for sleep quality across five occasions, *BHi* between-person random intercepts for happiness across five occasions. ^*^P < 0.05, ^†^*P* < 0.01, ^‡^*P* < 0.001

**Fig. 4:**
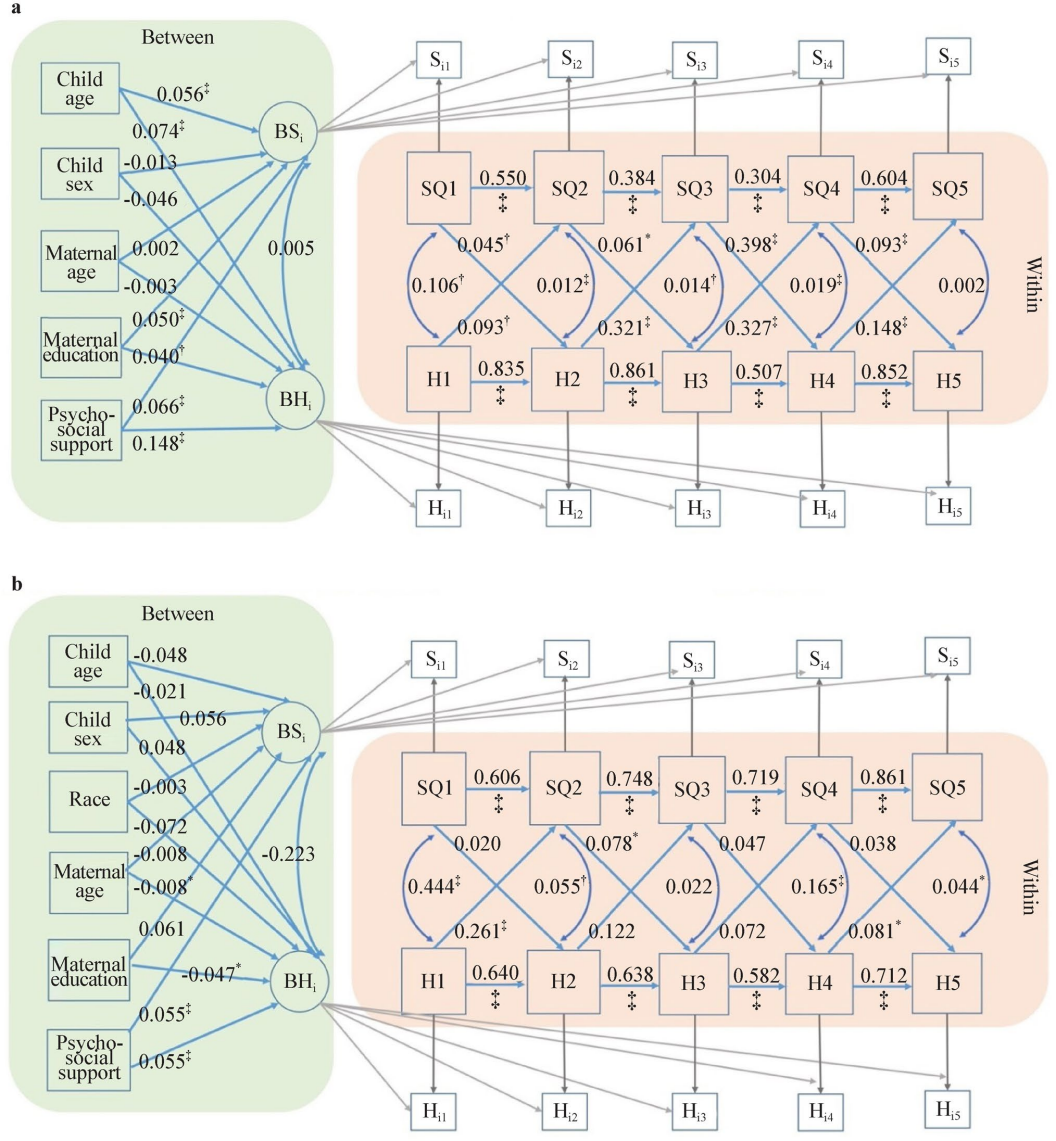
Random intercepts cross-lagged panel modeling with between-person covariates for the CJCC (**a**) and HBBS (**b**). *HBBS* Healthy Brain and Behavior Study, *CJCC* China Jintan Child Cohort, *SQ* sleep quality, *H* happiness, *BSi* between-person random intercepts for sleep quality across five occasions, *BHi* between-person random intercepts for happiness across five occasions. ^*^P < 0.05, ^†^*P* < 0.01, ^‡^*P* < 0.001

When the CJCC and HBBS cohorts were analyzed separately, the cross-lagged effects of happiness on sleep quality, and vice versa, were generally consistent between the combined cohort RI-CLPM models and the CJCC RI-CLPM models (Figs. 2 and 3). However, the cross-lagged effects in the HBBS cohort were less consistent. Specifically, significant cross-lagged effects of happiness on sleep quality were observed only from time 1 (0–1 year) to time 2 (1–3 years) and from time 4 (10–11 years) to time 5 (11 years to present). Similarly, significant cross-lagged effects of sleep quality on happiness were found only from time 2 (1–3 years) to time 3 (3–5 years) (*β* = 0.078, SE = 0.033, *P* < 0.01) (Fig. 4).

### Cohort comparison

**Fig. 5:**
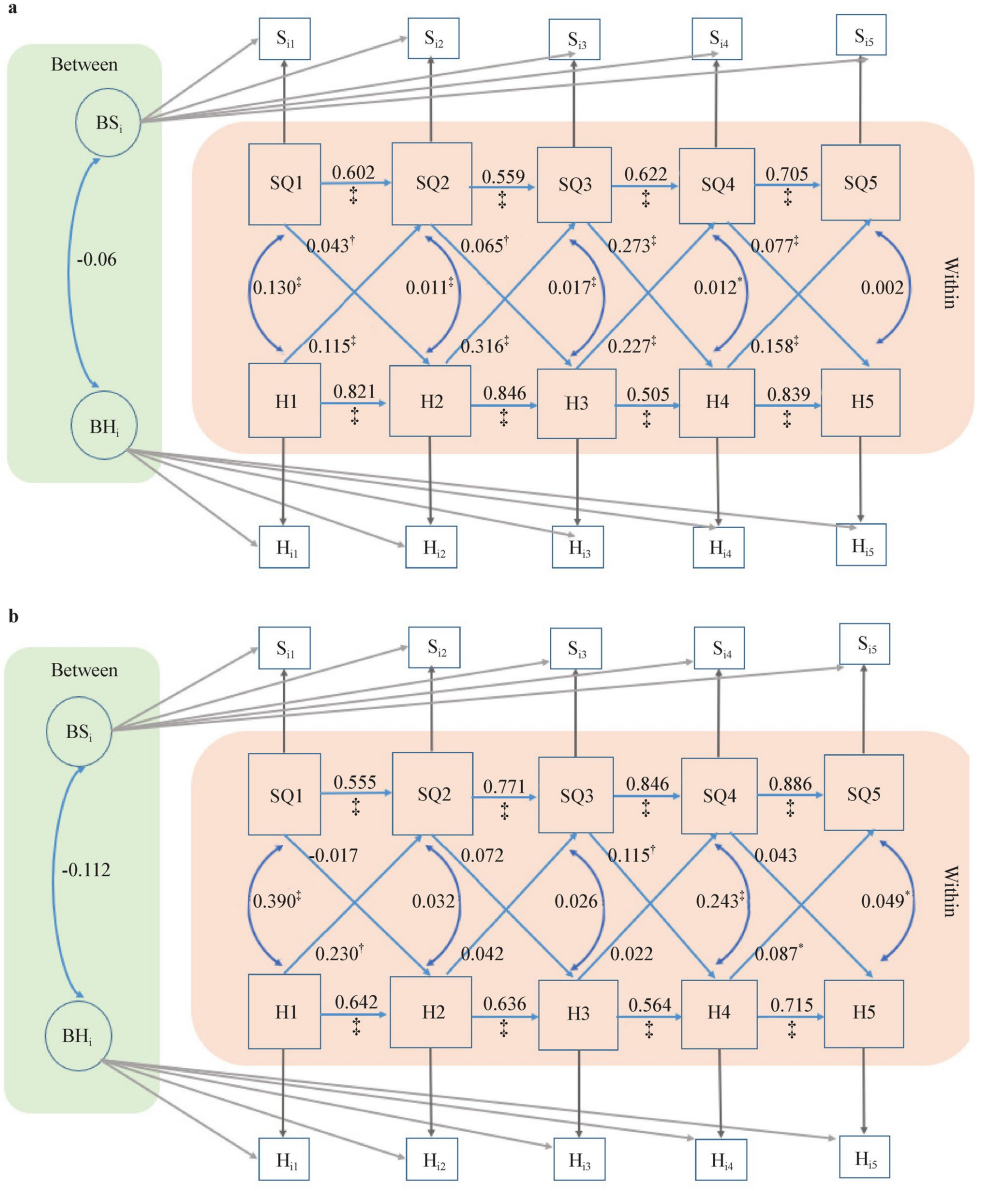
Multi-group random intercepts cross-lagged panel modeling without between-person covariates for the CJCC (**a**) and HBBS (**b**). *HBBS* Healthy Brain and Behavior Study, *CJCC* China Jintan Child Cohort, *SQ* sleep quality, *H* happiness, *BSi* between-person random intercepts for sleep quality across five occasions, *BHi* between-person random intercepts for happiness across five occasions. ^*^P < 0.05, ^†^*P* < 0.01, ^‡^*P* < 0.001

**Fig. 6:**
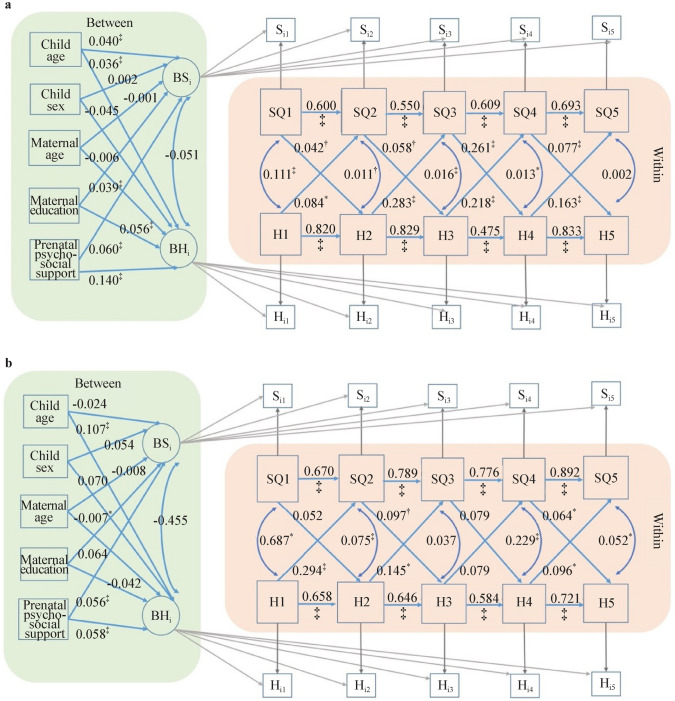
Multi-group random intercepts cross-lagged panel modeling with between-person covariates for the CJCC (**a**) and HBBS (**b**). *HBBS* Healthy Brain and Behavior Study, *CJCC* China Jintan Child Cohort, *SQ* sleep quality, *H* happiness, *BSi* between-person random intercepts for sleep quality across five occasions, *BHi* between-person random intercepts for happiness across five occasions. ^*^P < 0.05, ^†^*P* < 0.01, ^‡^*P* < 0.001

Multi-group analysis was conducted to assess the equivalence of the reciprocal relationship between happiness and sleep quality between the CJCC and the HBBS. The Chi-square difference test revealed a significant difference between CJCC and the HBBS regarding the relationship between happiness and sleep quality, Δχ^2^(*df* = 16) = 99.2, *P* < 0.001. Specifically, the cross-lagged effects of happiness on sleep quality and vice versa remained statistically significant in the CJCC across the five occasions, controlling for the autocorrelation effects and the concurrent within-person correlation (Fig. 5). However, in the HBBS, the cross-lagged effects of happiness on sleep quality remain statistically significant only from time 1 to time 2 (i.e., an increase in happiness at 0–1 year predicts an increase in sleep quality at 1–3 years) and from time 4 to time 5 (i.e., an increase in happiness at 5–11 years predicts an increase in sleep quality at 11 years–present), and the cross-lagged effects of sleep quality on happiness are statistically significant only at time 3 to time 4 (i.e., an increase in sleep quality at 3–5 years predicts an increase in happiness at 5–11 years). After adjusting for mother‒child dyads’ demographic characteristics, the statistical significance of the relationship between happiness and sleep quality remained the same in the two cohorts (Fig. 6).

### Sensitivity analysis

Using the non-imputed data, we found that the cross-lagged effects remained largely consistent with the analysis results from the imputed data, supporting the robustness of using imputed data to develop the RI-CLPM models for the two cohorts. The detailed modeling results for the non-imputed data are presented in Supplementary Table 2.

## Discussion

Using data from two cohorts, this study aimed to explore the long-term bidirectional relationship between sleep quality and happiness in children, examine whether this bidirectional relationship varies between the Chinese context and the U.S. context, and assess the predictive role of maternal psychosocial support during pregnancy in this relationship. The combined cohort analysis revealed a bidirectional, cross-lagged relationship between sleep quality and happiness from childhood to early adolescence (at ages 10–12). When the two cohorts were analyzed separately, this bidirectional relationship remained statistically significant across all developmental stages in the CJCC cohort but was only partially supported in the HBBS cohort. Maternal psychosocial support during pregnancy was a significant predictor of children’s sleep quality and happiness across different developmental stages, as demonstrated in both the combined cohort analysis and each cohort’s separate analysis.

### Children’s happiness and sleep quality over time

Our study revealed that children’s happiness and sleep quality developmental trajectories were relatively different between Chinese and U.S. children. With respect to the happiness development trajectory, Chinese children in the CJCC experienced a stable yet minor decrease in their happiness scores over time, whereas American children in the HBBS experienced a slight increase in their happiness score from infancy to toddlerhood, followed by a decrease after three years of age. The overall decreasing trend in happiness from early childhood to early adolescence was consistent with previous findings that young children generally remain happy and then become less happy when they enter ten years of age or even earlier in some countries [39, 40]. The differences in the happiness trajectory between the two cohorts can likely be attributed to the sociocultural context that shapes parent‒child dyads’ perspectives toward happiness [40]. Specifically, the differences in happiness trajectories between Chinese and U.S. children may be influenced by varying parental expectations, cultural values, educational systems, socialization practices, and economic and environmental factors, all of which shape how happiness is perceived, expressed, and experienced in each cultural context.

Regarding the sleep quality trajectory, we found that both Chinese and U.S. children’s average sleep quality increased from infancy to five years, which is similar to the results of a previous study [41]. A potential reason for this improvement is that sleep regulation is typically developed by the end of infancy and will further strengthen afterwards [42]. Specifically, from infancy to preschool age, children’s night-waking frequency generally decreases, whereas their nighttime sleep duration increases as they grow up [43]. Since night-waking frequency and sleep duration are objective indicators of one’s sleep quality [44], an improvement in these two indicators may make parents feel that their children’s sleep quality improves as they grow up. After five years of age, the average sleep quality of American children in the HBBS decreased with age. Potential reasons for this decreasing trend include adolescents’ nighttime social media use and psychological factors such as loneliness and anxiety [45, 46]. Another possible reason for the discrepancy in sleep quality trajectory between the two cohorts consists of cultural factors such as sleeping arrangements, school start time, and lifestyles [47].

### Bidirectional cross-lagged relationships between sleep quality and happiness

Combined cohort analysis revealed statistically significant yet modest cross-lagged effects between happiness and sleep quality. The standardized coefficients ranged from 0.047 to 0.188, suggesting an overall consistent yet subtle bidirectional relationship. While statistically significant, these small effect sizes indicate that happiness and sleep quality are interconnected but not strongly or deterministically linked. This finding is in line with the latest systematic review in that the daily affective state is bidirectionally associated with sleep [15]. Furthermore, our study extends this body of knowledge by showing that the bidirectional relationship between happiness and sleep quality starts in early childhood and lasts through early adolescence. Although the individual time-point effects are modest, the cumulative impact across developmental stages is noteworthy. The consistent directionality of these effects suggests that small, repeated interactions between happiness and sleep quality can meaningfully contribute to long-term psychological and physiological well-being.

An interesting pattern emerged from our findings where positive cross-lagged effects between sleep quality and happiness persisted despite their opposing developmental trajectories. Happiness showed an overall declining trend in both cohorts, whereas sleep quality demonstrated an increasing trend in China’s CJCC but a decreasing trend in the U.S.’s HBBS. Nevertheless, the bidirectional relationship between sleep quality and happiness remained positive. This pattern suggests that although the absolute levels of these variables may follow different developmental paths, their relative changes still positively influence one another. For example, even though average sleep quality may decline with age, children who maintain relatively better sleep quality than their peers are more likely to experience increased happiness in subsequent periods. Similarly, children who report relatively higher happiness levels are more likely to experience better sleep quality in subsequent periods, even though the overall trend in sleep quality declines. This distinction between absolute trajectories and relative influences underscores the complexity of developmental processes, where overall age-related trends can coexist with positive reciprocal relationships between variables.

Regarding the cross-country comparison of the bidirectional relationship, we found that within-person happiness fluctuations positively predicted subsequent sleep quality fluctuations, and this directional relationship was consistently found both in Chinese children and in American children. This finding is similar to that of a recent study that investigated the bidirectional relationship between daily mood and nightly sleep in adolescents [48]. In their study, Kouros and colleagues reported daytime happiness as both a between-person and within-person level predictor of fewer self-reported sleep/wake problems during the same night, meaning that, on average, adolescents who were happier during the daytime reported fewer sleep/wake problems at night, and higher than usual daytime happiness in adolescents was also associated with fewer self-reported sleep/wake problems that night [48]. Another study reported that adolescents with lower subjective happiness also reported poor sleep quality [18]. A potential mechanism for this directional effect is that children with higher levels of happiness are more likely to engage in healthy behaviors and have good sleep hygiene [14]. Another possible reason is that children with higher levels of happiness may be more resilient and thus can more effectively cope with stressful events that may hinder sleep quality.

However, the cross-lagged effects of sleep quality on happiness were less consistent between the Chinese and U.S. contexts. The stronger associations observed in the Chinese cohort (CJCC) than in the U.S. cohort (HBBS) suggest potential cultural moderating effects. The effect size differences highlight the importance of sociocultural context in understanding child developmental trajectories. This finding in the American sample is consistent with that reported by Kouros et al., who reported that the within-person relationship between sleep/wake problems and happiness the next day was not significant among American adolescents in a 7-day ecological momentary assessment study [46]. One plausible reason is that the happiness of American children in the HBBS may be more influenced by factors other than their sleep quality. A comparison of life events during pregnancy between CJCC mothers and HBBS mothers revealed that HBBS mothers faced greater social adversity, including higher rates of divorce or separation, unemployment, moving, financial problems, and conflict with significant others. These factors have been shown in the United Nations Children’s Fund’s report to have a negative effect on children’s well-being and life satisfaction in rich countries [49]. Thus, whether sleep quality has a greater impact on happiness for children with better family environments warrants future investigation. Another potential reason for this discrepancy between the two contexts is the influence of socioeconomic status (SES) on children’s sleep quality and happiness. Although several SES factors were collected in both cohorts, direct comparisons between them were challenging due to the distinct economic contexts and measurement approaches used in Philadelphia and Jintan cities in China. Differences in income levels, educational standards, and indicators of social status operate uniquely in these two settings, complicating standardized SES comparisons. Furthermore, cultural differences in sleep practices, such as cosleeping arrangements, bedtime routines, and parental expectations about sleep, may also influence how U.S. and Chinese mothers perceive and report their children’s sleep quality [50, 51]. Future research could benefit from incorporating standardized, objective sleep measurements and detailed documentation of sleep behaviors to gain a better understanding of cross-cultural differences in children’s sleep patterns.

### Maternal psychosocial support during pregnancy

Regardless of the minor inconsistencies in the bidirectional relationship between the two contexts, maternal psychosocial support during pregnancy was consistently found to be predictive of higher happiness and better sleep quality from infancy to early adolescence. Social support during pregnancy serves as a protective factor against stress, anxiety, and depression in expectant mothers, which are known to adversely affect fetal development and can have long-term implications for the child’s emotional and physical health [52]. Indeed, women who have lower psychosocial support during pregnancy may have greater perceived stress, depression, negative spousal support and low self-efficacy [53]. These demographic and social characteristics are not easily ameliorated and are likely to last beyond pregnancy [54] and increase the cumulative risk of children’s sleep problems [55] and subjective well-being. Additionally, given that maternal reports were used to assess both psychosocial support and their child’s happiness and sleep quality, another potential explanation for the observed predictive effects is that mothers with low social support, who experienced elevated stress and depressive moods during pregnancy, may have a generally negative outlook, which could lead to perceiving their children as having lower happiness and poorer sleep health.

### Limitations

This study has several limitations. First, both children’s happiness and sleep quality were reported by their mothers, which may introduce recall bias and reflect maternal perceptions of their children’s mental and sleep health, so the results should be treated with caution. Second, both children’s happiness and sleep quality were measured with a single question, which may not fully reflect the true state or nuances of children’s happiness and sleep health. However, studies have shown that parent-reported child sleep outcomes are generally comparable to objectively measured sleep variables and can reflect certain aspects of children’s sleep health [56, 57]. With respect to happiness scores, the majority of studies continue to use a single item to rate adolescent happiness [58]. In addition, measuring happiness with a single item has been demonstrated in previous research to be reliable, valid, and viable in cross-cultural comparisons [59]. Furthermore, several factors should be considered when interpreting the generalizability of our findings. The U.S.’s HBBS sample primarily represents urban and inner-suburban African American communities in Philadelphia (80.3% African American), rather than the broader U.S. population. While this provides valuable insights into child development within this specific community context, the findings may not be generalizable to other populations or settings in the U.S.. Similarly, while China’s CJCC sample was demographically representative of Jintan city through stratified random sampling of preschools across different socioeconomic strata, it reflects the context of a medium-sized eastern Chinese city. It may not capture the diversity of environments across China, particularly those in major metropolitan areas or rural regions.

Our findings highlight significant associations between prenatal psychosocial support and children’s happiness and sleep quality trajectories. However, these relationships should be interpreted as correlational rather than causal, suggesting potential confounding factors. For example, mothers who report greater prenatal support may also provide more nurturing postnatal environments, maintain better mental health throughout their children’s development, or have greater access to social and economic resources, all of which could independently influence children’s happiness and sleep quality. Despite these limitations, this study has strengths in that it is the first study to investigate the long-term bidirectional relationship between happiness and sleep quality from children’s developmental perspective and to compare this bidirectional relationship between two different cultural contexts.

In conclusion, this study highlights an overall bidirectional relationship between happiness and sleep quality from infancy to early adolescence. The finding that happiness predicts better subsequent sleep quality is more consistent across the Chinese and U.S. contexts than in the reverse direction. Maternal psychosocial support during pregnancy emerged as a consistent protective factor for children’s happiness and sleep quality across multiple developmental ages.

These findings underscore the importance of integrating healthcare services with broader social policies to promote equitable access to resources and psychosocial support systems for pregnant women. Psychosocial support should be viewed not just as individual care but also as a collaborative effort across healthcare, education, and social service sectors to foster a supportive environment for families. Future research should investigate the bidirectional relationship between children’s happiness and objective sleep measures while examining this dynamic across cultural contexts.

## Supplementary Information

Below is the link to the electronic supplementary material.

Supplementary file 1 (PDF 469 KB)

## Data Availability

The data are not publicly available due to ethical restrictions but are available from the corresponding author on reasonable request.
