# Supplementary material for: Bidirectional relationship between child happiness and sleep quality and the predictive role of prenatal psychosocial support: results from U.S. and China cohort studies
Source: World J Pediatr. 2025 May 30;21(11):1102–19. doi: 10.1007/s12519-025-00903-9 (PMC12627121; doi:10.1007/s12519-025-00903-9)
Supplement: Supplementary file 1 — (PDF 469 KB) [file 12519_2025_903_MOESM1_ESM.pdf]

Supplementary materials

Supplementary Table 1. Mixed-effects linear models of happiness and sleep quality trajectory using two cohorts' combined dataset

Parameters	Happiness		Sleep quality	
	Model 1	Model 2	Model 3	Model 4
	β (SE)	β (SE)	β (SE)	β (SE)
Fixed effects				
Intercept ($\beta 0$)	4.751 (0.017)‡	4.763 (0.028)‡	3.203 (0.022)‡	3.028 (0.037)‡
Age ($\beta 1$)	-0.030 (0.002)*	-0.047 (0.003)‡	0.026 (0.002)‡	-0.006 (0.004)
Cohort ($\beta 2$)		-0.019 (0.035)	-0.162 (0.098)	0.269 (0.046)‡
Age*cohort ($\beta 3$)		0.025 (0.004)‡		0.049 (0.005)‡
Variance components				
Level-1: within-person ($\sigma\varepsilon$^2)	0.09857	0.09856	0.15973	0.1597
Level-2: in intercept ($\sigma 0$^2)	0.30196	0.30189	0.56215	0.5457
Level-2: in rate of change ($\sigma 1$^2)	0.00347	0.00332	0.00645	0.0059
Goodness-of-fit				
Deviance	8545	8484	11,972	11,641
AIC	8557	8500	11,984	11,657
BIC	8598	8554	12,025	11,711

AIC Akaike information criterion, *BIC* Bayesian information criterion, *SE* standard error. $^{*}P < 0.05$, $^{†}P < 0.01$, $^{‡}P < 0.001$

Supplementary Table 2. Random-intercepts cross-lagged modeling of child happiness and child sleep quality trajectory using un-imputed data

Variables	CJCC + HBBS		CJCC		HBBS	
	Model 1	Model 1a	Model 2	Model 2a	Model 3	Model 3a
	β (SE)	β (SE)	β (SE)	β (SE)	β (SE)	β (SE)
**Autoregressive effects**						
Happiness at 0-1 y→happiness at 1-3 y (alpha2)	0.714 (0.042)‡	0.679 (0.044)‡	0.773 (0.058)‡	0.688 (0.071)‡	0.614 (0.053)‡	0.613 (0.055)‡
Happiness at 1-3 y→happiness at 3-5 y (alpha3)	0.753 (0.042)‡	0.723 (0.048)‡	0.829 (0.055)‡	0.831 (0.058)‡	0.691 (0.064)‡	0.635 (0.071)‡
Happiness at 3-5 y→happiness at 5-11 y (alpha4)	0.560 (0.054)‡	0.545 (0.059)‡	0.340 (0.132)†	0.183 (0.138)	0.593 (0.065)‡	0.557 (0.069)‡
Happiness at 5-11 y→happiness at 11 y-present (alpha5)	0.783 (0.030)‡	0.776 (0.030)‡	0.932 (0.044)‡	0.923 (0.048)‡	0.726 (0.039)‡	0.723 (0.039)‡
Sleep quality at 0-1 y→sleep quality at 1-3 y (delta2)	0.608 (0.056)‡	0.641 (0.056)‡	0.501 (0.045)‡	0.491 (0.050)‡	0.568 (0.066)‡	0.614 (0.067)‡
Sleep quality at 1-3 y→sleep quality at 3-5 y (delta3)	0.755 (0.050)‡	0.768 (0.049)‡	0.481 (0.055)‡	0.557 (0.064)‡	0.724 (0.061)‡	0.750 (0.069)‡
Sleep quality at 3-5 y→sleep quality at 5-11 y (delta4)	0.763 (0.058)‡	0.756 (0.061)‡	0.525 (0.120)‡	0.601 (0.115)‡	0.691 (0.072)‡	0.708 (0.073)‡
Sleep quality at 5-11 y→sleep quality at 11 y-present (delta5)	0.877 (0.028)‡	0.879 (0.030)‡	0.672 (0.068)‡	0.683 (0.075)‡	0.871 (0.034)‡	0.873 (0.035)‡
**Cross-lagged effects**						
Happiness at 0-1 y→sleep quality at 1-3 y (gamma2)	0.276 (0.065)‡	0.271 (0.068)‡	0.287 (0.075)‡	0.204 (0.092)*	0.280 (0.080)‡	0.257 (0.084)†
Happiness at 1-3 y→sleep quality at 3-5 y (gamma3)	0.150 (0.056)†	0.158 (0.060)†	0.410 (0.061)‡	0.312 (0.070)‡	0.129 (0.080)	0.100 (0.082)
Happiness at 3-5 y→sleep quality at 5-11 y (gamma4)	0.111 (0.055)*	0.113 (0.060)	0.297 (0.089)‡	0.137 (0.090)	0.101 (0.075)	0.085 (0.079)
Happiness at 5-11 y→sleep quality at 11 y-present (gamma5)	0.102 (0.032)‡	0.088 (0.033)†	0.185 (0.061)†	0.157 (0.068)*	0.086 (0.041)*	0.070 (0.042)
Sleep quality at 0-1 y→happiness at 1-3 y (beta2)	0.035 (0.025)	0.051 (0.024)*	0.079 (0.028)†	0.064 (0.030)*	0.023 (0.031)	0.030 (0.031)
Sleep quality at 1-3 y→happiness at 3-5 y (beta3)	0.085 (0.025)‡	0.080 (0.025)‡	0.108 (0.041)†	0.065 (0.036)	0.057 (0.036)	0.078 (0.036)*
Sleep quality at 3-5 y→happiness at 5-11 y (beta4)	0.143 (0.040)‡	0.094 (0.043)*	0.411 (0.129)‡	0.297 (0.123)*	0.043 (0.047)	0.044 (0.048)
Sleep quality at 5-11 y→happiness at 11 y-present (beta5)	0.066 (0.023)†	0.061 (0.022)†	0.048 (0.042)	0.023 (0.037)	0.040 (0.030)	0.048 (0.029)
**Concurrent covariance between sleep quality and happiness at the following age (y)**						
0-1	0.504	0.492	0.296	0.126	0.465	0.460

	(0.182)†	(0.174)†	(0.146) *	(0.064)*	(0.138)‡	(0.141)‡
1-3	0.052 (0.014)‡	0.060 (0.013)‡	0.044 (0.010)‡	0.041 (0.011)‡	0.060 (0.020)†	0.065 (0.020)‡
3-5	0.024 (0.011)*	0.021 (0.011)	0.022 (0.007)†	0.014 (0.006)*	0.019 (0.020)	0.018 (0.020)
5-11	0.155 (0.021)‡	0.154 (0.023)‡	0.071 (0.015)‡	0.057 (0.015)‡	0.174 (0.036)‡	0.175 (0.037)‡
11-present	0.029 (0.011)†	0.029 (0.011)†	0.004 (0.005)	0.011 (0.005)*	0.042 (0.020)*	0.035 (0.020)
**Variance of random intercepts**						
Happiness (ix)	0 (0.190)	0 (0.091)	0.007 (0.142)	0.139 (0.053)†	0 (0.076)	0 (0.072)
Sleep quality (iy)	0 (0.333)	0 (0.358)	0 (0.150)	0.010 (0.081)	0 (0.279)	0 (0.309)
**Covariance of two random intercepts**	-0.298 (0.177)	-0.294 (0.167)	-0.154 (0.139)	-0.021 (0.056)	-0.199 (0.128)	-0.218 (0.131)
**Between-person predictors on random intercepts**						
Child age at survey						
Child age at survey→random intercepts of happiness (ix)		-0.031 (0.033)		-0.002 (0.058)		-0.031 (0.038)
Child age at survey→random intercepts of sleep quality (iy)		-0.027 (0.047)		-0.013 (0.052)		-0.027 (0.069)
Gender						
Gender→random intercepts of happiness (ix)		-0.003 (0.038)		-0.081 (0.066)		0.028 (0.045)
Gender→random intercepts of sleep quality (iy)		0.055 (0.055)		-0.002 (0.059)		0.086 (0.081)
Maternal education						
Maternal education→random intercepts of happiness (ix)		-0.006 (0.020)		0.026 (0.032)		-0.035 (0.024)
Maternal education→random intercepts of sleep quality (iy)		0.038 (0.028)		0.018 (0.029)		0.055 (0.044)
Maternal age when child was born						
Maternal age→random intercepts of happiness (ix)		-0.007 (0.004)		-0.004 (0.011)		-0.005 (0.004)
Maternal age→random intercepts of sleep quality (iy)		-0.003 (0.005)		0.018 (0.010)		-0.005 (0.006)
Maternal psychosocial support during pregnancy						

Psychosocial support→random intercepts of happiness (ix)		0.059 (0.008)‡		0.102 (0.022)‡		0.051 (0.008)‡
Psychosocial support→random intercepts of sleep quality (iy)		0.052 (0.012)‡		0.054 (0.020)†		0.052 (0.014)‡
Race						
Race→random intercepts of happiness (ix)						-0.089 (0.058)
Race→random intercepts of sleep quality (iy)						0.007 (0.105)
Cohort						
Cohort→random intercepts of happiness (ix)		-0.001 (0.061)				
Cohort→random intercepts of sleep quality (iy)		0.718 (0.089)‡				
**Model fit indices**						
CFI	0.989	0.975	0.986	0.985	0.988	0.979
TLI	0.978	0.962	0.972	0.977	0.976	0.969
RMSEA	0.055	0.048	0.074	0.047	0.052	0.040
SRMR	0.028	0.053	0.034	0.033	0.027	0.031
**Number of observations**	740	671	304	265	436	406

HBBS Healthy Brain and Behavior Study, *CJCC* China Jintan Child Cohort, *CFI* comparative fit index, *TLI* Tucker–Lewis index, *RMSEA* root-mean-square error of approximation, *SRMR* standardized root-mean-square residual

The common method bias and sensitivity analysis

Given that child happiness and sleep quality were assessed by mothers using a one-item scale, there is a potential risk of common method bias (CMB). To detect CMB, we conducted Harman's single factor analysis and an unmeasured latent method construct analysis following the suggestions from Kock and colleagues [1]. The Harman's single factor analysis showed that the total variance explained by the one-factor model was only 32%, suggesting that CMB might not be a significant issue. However, the unmeasured latent factor model showed improvements in fit indices such as the comparative fit index, Tucker–Lewis index, root-mean-square error of approximation, and standardized root-mean-square residual, and notable differences in standardized path coefficients between baseline model and the latent factor CFA model, indicating that CMB may indeed present in our baseline model. To

mitigate this issue, we performed sensitivity analyses using adolescent self-reported happiness measured by the Oxford happiness inventory and sleep quality assessed by the Pittsburgh sleep quality index when the China Jintan Child Cohort adolescents were 10-13 years old.

Reference

[1] Kock F, Berbekova A, Assaf AG. Understanding and managing the threat of common method bias: detection, prevention and control. Tour Manag. 2021;86:104330.

Supplementary Figures

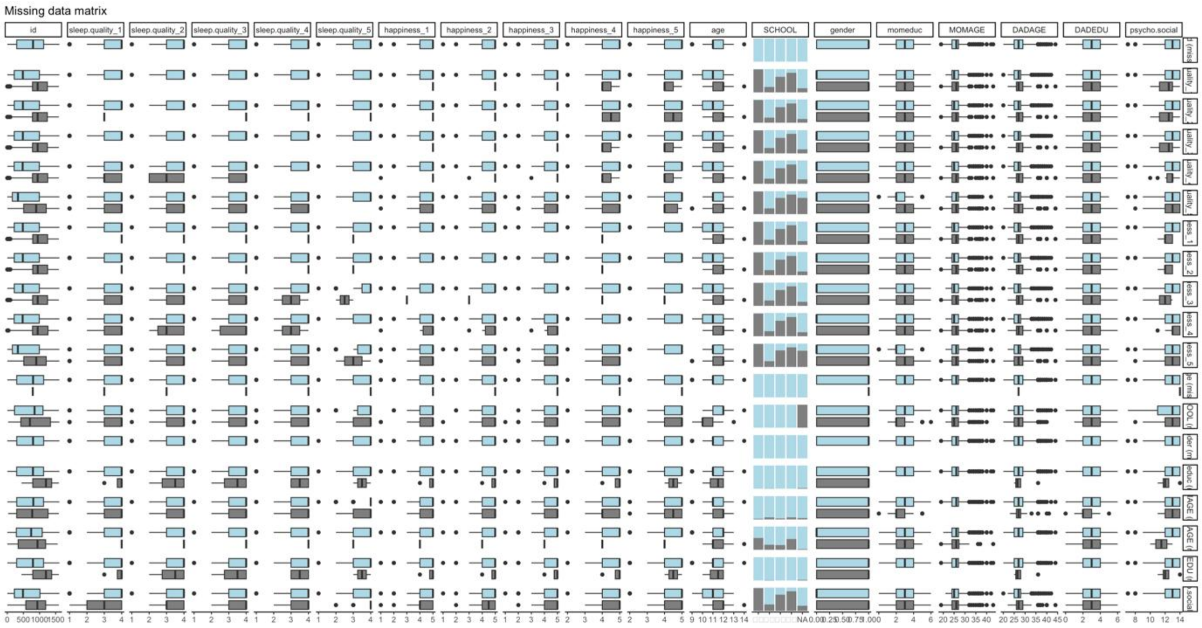

Supplementary Fig. 1 Missing data pattern for China Jintan Child Cohort

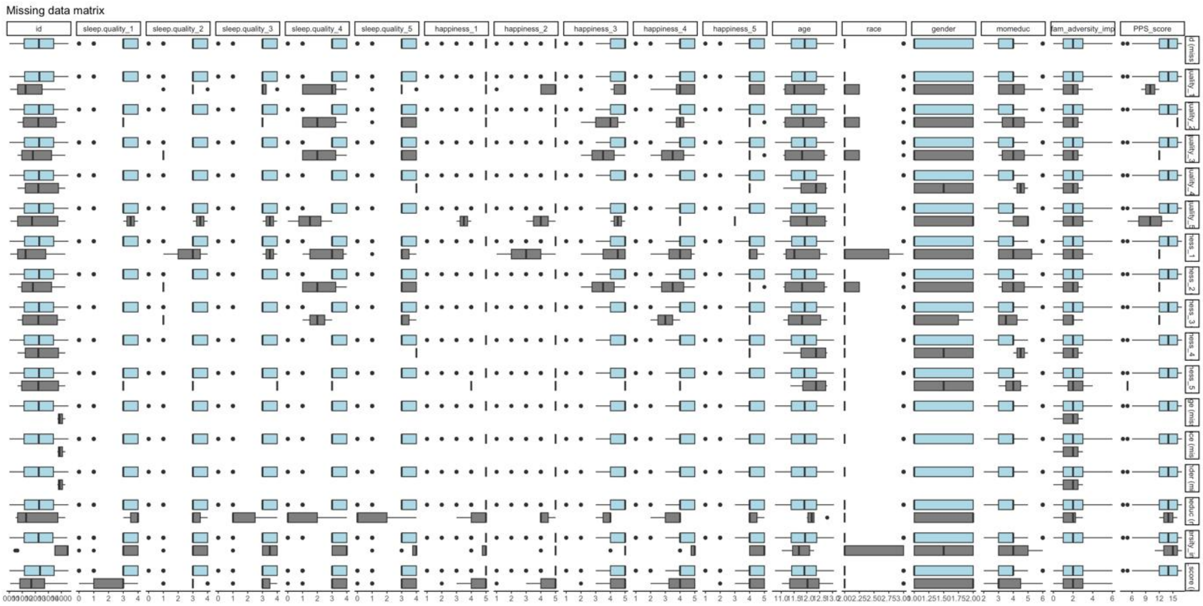

Supplementary Fig. 2 Missing data pattern for Healthy Brain and Behavior Study
